# Evaluation of Mesh Closure of Laparotomy and Extraction Incisions in Open and Laparoscopic Colorectal Surgery: A Systematic Review and Meta-Analysis †

**DOI:** 10.3390/jcm13226980

**Published:** 2024-11-20

**Authors:** Mohamed Albendary, Ali Yasen Mohamedahmed, Marwa Yassin Mohamedahmed, Ugochukwu Ihedioha, Shantanu Rout, Anouk Van Der Avoirt

**Affiliations:** 1 General Surgery Department, Northampton General Hospital NHS Trust, Northampton NN1 5BD, UK; 2 Brighton and Sussex Medical School, University of Brighton, Brighton BN1 9PX, UK; a.vanderavoirt@bsms.ac.uk; 3 General Surgery Department, University Hospitals of Derby and Burton NHS Trust, Derby DE22 3ND, UK; dr.aliyasen1@gmail.com; 4 General Surgery Department, Atbara Teaching Hospital, Atbara PX3H+822, Sudan; marwa.y.mohamed@outlook.com; 5 General Surgery Department, Sandwell and West Birmingham NHS Trust, West Bromwich B71 4HJ, UK; 6 University Hospitals Sussex NHS Trust, Worthing BN2 5BE, UK

**Keywords:** prophylactic mesh, colorectal surgery, incisional hernia

## Abstract

**Background and Objectives**: Evisceration and incisional hernia (IH) represent a significant morbidity following open or laparoscopic colorectal surgery where midline laparotomy or extraction incision (EI) are performed. We executed a systematic review to evaluate primary mesh closure of laparotomy or EI in colorectal resections of benign or malignant conditions. **Methods**: A comprehensive literature search was performed using PubMed, Science Direct, Cochrane, and Google Scholar databases for studies comparing prophylactic mesh to traditional suture techniques in closing laparotomy in open approach or EI when minimally invasive surgery was adopted in colorectal procedures, regardless of the diagnosis. Both IH and evisceration were identified as primary outcomes. Secondary outcomes included surgical site infections (SSI), postoperative seroma, and length of hospital stay (LOS). **Results**: Six studies were included in our analysis with a total population of 1398 patients, of whom 411 patients had prophylactic mesh augmentation when closing laparotomy or EI, and 987 underwent suture closure. The mesh closure group had a significantly lower risk of developing IH compared to the conventional closure group (OR 0.23, *p* = 0.00001). This result was significantly consistent in subgroup analysis of open laparotomy or EI of laparoscopic surgery subgroups. There was no statistically notable difference in evisceration incidence (OR 0.51, *p* = 0.25). Secondary endpoints did not significantly differ between both groups in terms of SSI (OR 1.20, *p* = 0.54), postoperative seroma (OR 1.80, *p* = 0.13), and LOS (MD −0.54, *p* = 0.63). **Conclusions:** primary mesh reinforcement of laparotomy or EI closure in colorectal resections lessens IH occurrence. No safety concerns were identified; however, further high-quality research may provide more solid conclusions.

## 1. Introduction

An incisional hernia (IH) is a protrusion of the abdominal content through a defect in the weakened abdominal wall at the site of a previous surgical incision. It is reported as 10–40% following elective laparotomy and even higher in emergency settings, reaching up to 54% [1,2]. Recurrence, with a rate of around 20%, accounts for a relatively large proportion of all incisional hernias [3]. Previous surgical incisions, wound infection, increased tissue tension, and connective tissue disorders contribute to impaired healing and subsequent hernia formation. In high-risk populations, such as those with obesity, smoking, connective tissue disorder, and neoplastic conditions, IH is more prevalent [4].

Colorectal procedures come with a relatively higher IH risk, with an estimate of 31.5% in a 2-year follow-up [5]. These hernias develop not only in the main laparotomy incision but also in the extraction incision (EI), which is used to extract specimens in the laparoscopic approach [6]. The combination of the clean-contaminated or contaminated nature and patients’ comorbidities increases the risk of SSI, which is a common cause of IH occurrence. 

Not only does IH have a significant impact on the quality of life of the patients, but it also impacts healthcare systems as a financial burden and service overload [7,8]. Patients with symptomatic IH have unsatisfactory cosmetic appearance, limited physical activity, and impaired quality of life with serious life-threatening disorders, including incarceration and bowel strangulation [9]. The cost of 6.3 billion US dollars, estimated for approximately 89,258 IH repair procedures annually in the US, significantly impacts financial resources [10]. It is estimated that 82% of the hernias repaired in England are incisional hernias [11]. Also, the high recurrence rate represents an unfavourable factor, and certain high-risk patients may not be offered this second chance due to morbidity and recurrence [12].

An estimate of 4 million Euros is achievable with a 5% reduction in IH occurrence through optimising abdominal closure techniques and modifiable risk factors in patients [8]. Hence, focusing on preventing IH occurrence remains the key to lessening morbidity and costs. The prophylactic use of mesh is a potentially effective way to reduce the incidence of IH, especially in high-risk patients [13]. This systematic review evaluates the efficacy of prophylactic mesh in reducing the IH rate following colorectal surgery and any potential adverse events.

## 2. Materials and Methods

This systematic review was designed, performed, and reported as per the recommendations of the Cochrane Handbook for Systematic Reviews of Interventions and Preferred Reporting Items for Systematic Reviews and Meta-Analyses (PRISMA) guidelines [14,15]. The PRISMA checklist is provided in Supplementary S1. The abstract of this review has been presented in an international conference in May 2024 [16].

Studies included in this analysis were based on the following PICOS (Population, Intervention, Comparator, Outcomes, Study design):

P: Patients undergoing open or laparoscopic colorectal operations. 

I: Closure of laparotomy or specimen extraction incisions with mesh.

C: Conventional closure (without mesh) of laparotomy or specimen extraction incisions.

O: Rate of IH occurrence at the maximum follow-up period and early (in 30 days postoperatively), postoperative complications, length of hospital stay (LOS).

S: Systematic review and meta-analysis of comparative studies. 

Inclusion criteria and exclusion criteria

The inclusion criteria for studies were as follows:

- Randomised controlled trials (RCTs) or comparative studies.
- Studies including patients of all age groups and of any gender.
- Studies including patients who underwent colorectal surgery, either for benign or malignant conditions.
- Studies including patients who underwent laparotomy or laparoscopy with EI performed.
- Studies comparing mesh closure of laparotomy or EI versus conventional suture closure.
- Studies reporting IH and/or evisceration (wound dehiscence) as primary outcome.

The exclusion criteria for studies were as follows:

- Non-comparative studies.
- Studies of mixed surgeries, not only colorectal.
- Case series, case reports, and letters.

### 2.1. Search Strategy

A literature search was performed using the following databases: PubMed, Medline, Embase, Cochrane Library, and Scopus in December 2022. A second search was conducted in June 2024 to ensure the captured data from the literature were up to date. The search was limited to human objects, and no publishing dates or language restrictions were applied when searching available publications. The above-mentioned inclusion and exclusion criteria were followed. The references of related published reviews and eligible studies were also screened to recognise any potential studies. As for the keywords and search terms, they were combined as follows: (Mesh OR “prophylactic mesh” OR “Mesh closure”) AND (suture OR “suture closure” OR “conventional closure”) AND (“colorectal surgery” OR colon OR rectum).

### 2.2. Study Selection and Data Extraction

Two authors executed the literature search; however, a third author was consulted in cases of ambiguous research or unclear outcomes. As explained in the search strategy section, the search was conducted twice, and duplicated studies were excluded. Abstracts were screened for relevance and classified as included, excluded, or requiring further evaluation. All studies meeting the inclusion criteria were included. Any confronting data or unclear reports were addressed, and if needed, they were addressed by consulting a third author. Full texts of relevant studies were reviewed, and there was no missing information from the authors to enquire about. As per the PRISMA flowchart shown later on in the results, the eligible studies were included in this review, and the available outcomes were pooled and reported in the meta-analysis.

All data were extracted manually, revised, and recorded in a pre-created standard Microsoft^®^ Excel (Microsoft Corporation, Redmond, WA, USA) file. The information collected from each study was the first author, year of publication, country of origin, study design, number of populations in the study, mesh insertion arm or no-mesh arm, and comparative outcomes. Data on population characteristics, follow-up periods, and technical steps were also obtained.

### 2.3. Outcome Measures

The primary outcomes are the rate of IH occurrence at the maximum follow-up period and early (in 30 days postoperatively) postoperative evisceration. IH was diagnosed by clinical examination and/or imaging. Postoperative complications, including surgical site infection (SSI), seroma development, and length of hospital stay (LOS), were considered secondary outcomes.

### 2.4. Risk of Bias

The randomised controlled trials (RCTs) were evaluated for risk of bias using the Cochrane risk of bias tool [17]. This included reviewing the RCTs and grading the bias risk as “high”, “low” or “unclear” in the different aspects of random sequence generation, allocation concealment, blinding of participants, blinding of outcome assessment, incomplete outcome data, selective reporting, and other potential sources. Newcastle–Ottawa scale (NOS) was used to assess the risk of bias in the observational studies [18]. NOS is a point-based scoring system with a maximum score of 9, which assesses observational studies based on the following domains: selection of study groups, comparability of the groups, ascertainment of the proposed outcome, and follow-up of cohorts. Studies were considered low, medium, or high risk of bias if the NOS was 9, 7 or 8, or 6 and less, respectively.

### 2.5. Statistical Analysis

Pooling the data and the statistical analysis for the meta-analysis were executed using the RevMan Version 5.4 software. Dichotomous outcomes were compiled with a random effects model to measure odds ratios (OR) with a 95% confidence interval (CI). A mean difference (MD) with 95% CI was assessed for continuous outcomes. Mean standard deviation (SD) was estimated using the Hozo et al. equation when continuous variables were reported as median and interquartile range (IQR) [19]. Random effects modelling was implemented for all outcomes analysis.

The results were deemed statistically significant if the *p*-value was <0.05 or if the 95% CI did not include 1.00. The Cochran Q test (χ^2^) and I^2^ statistics were utilised to evaluate data heterogeneity. An I^2^ value exceeding 50% is associated with significant heterogeneity, while a value of 0% means no heterogeneity.

To evaluate the validity of the results, sensitivity analysis was performed by calculating the odds ratio for dichotomous variables. The primary analyses for outcome parameters were also repeated using a fixed-effects model. Moreover, a leave-one-out analysis was used to examine the impact of each study on the overall effect size and heterogeneity.

## 3. Results

**Figure 1:**
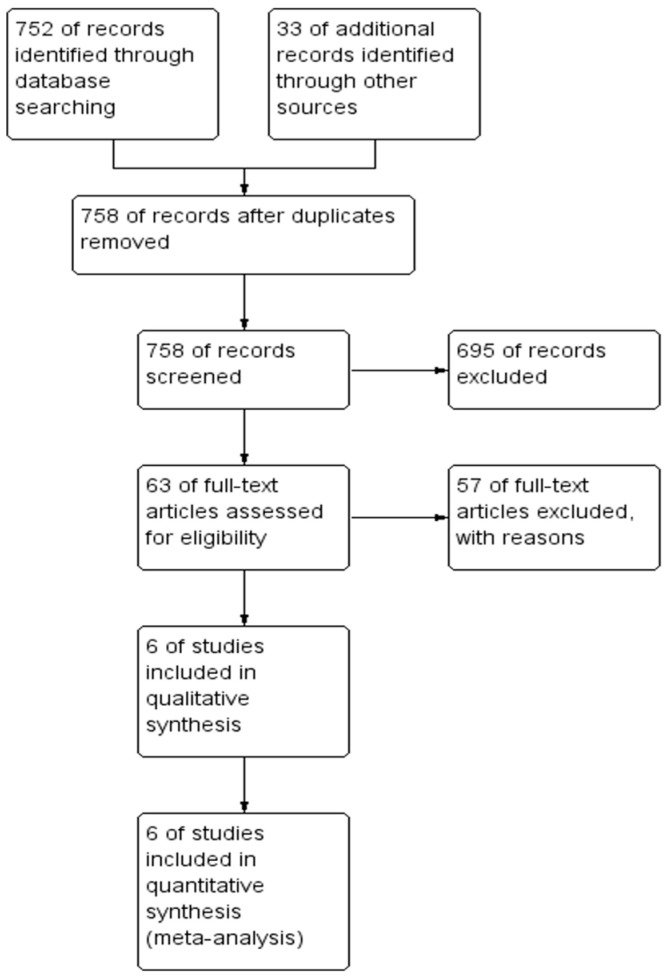
PRISMA flowchart.

The search identified 758 studies in total, of which 695 were excluded after reviewing titles and abstracts. On further evaluation of the full texts of the remaining 63 studies, 57 publications did not fulfil eligibility criteria. Hence, six studies [5,20,21,22,23,24] were included in the systematic review, and reported outcomes were pooled for a meta-analysis. The methodology section demonstrates the inclusion criteria, and the PRISMA flowchart is shown in Figure 1.

These six studies included 1398 patients, with 411 having prophylactic mesh insertion at the time of midline laparotomy or EI closure and 987 undergoing conventional suture closure. Table 1 demonstrates the characteristics of the included studies, surgical techniques, and follow-up.

A summary of all the pooled outcomes is provided in Table 2, for convenience.

### 3.1. Methodological Appraisal

The demographics and comorbidities of the patient population in the selected studies were vastly comparable, as detailed in Table 3.

As for mesh insertion, it was randomised in the included RCTs, whereas in comparative research, this allocation was either left to the surgeon’s discretion or restricted to high-risk patients. Two of the included studies are randomised controlled trials (RCTs) [5,20], and the other four are comparative studies [21,22,23,24]. The oncological diagnosis was reported as 100% in two studies [21,22] and overall dominant in the rest of the studies. The outcomes in cancer or non-cancer groups were not reported separately hence, any possible influence could not be examined. While four evaluated the use of mesh in laparotomy closure following open approach surgery, two studies examined mesh closure technique in EI of laparoscopic surgeries [21,22].

**Figure 2:**
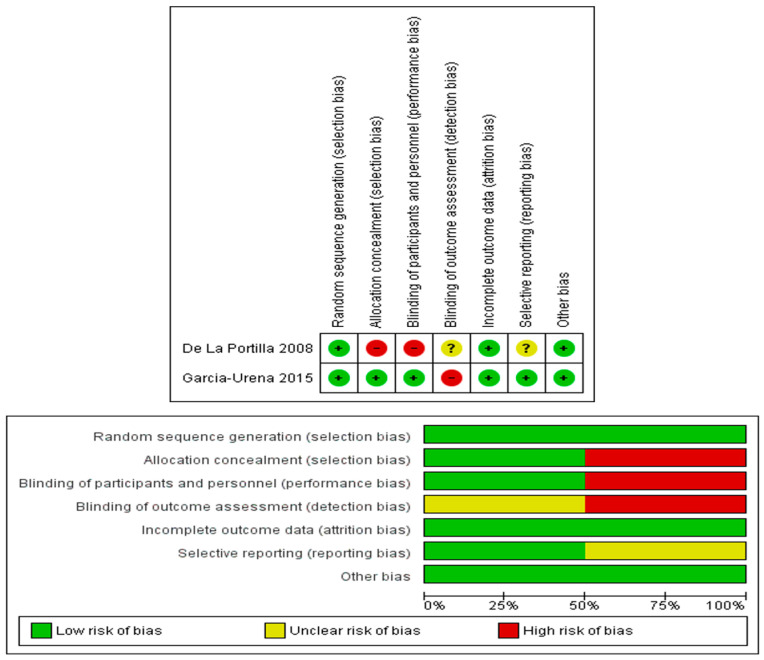
Cochrane risk of bias assessment for RCTs.

One of the RCTs is measured as high quality with only risk of detection bias [5], while the other has some concerns about selection and performance bias [20]. Three of the comparative studies were measured as medium risk [21,23,24]. On the other hand, the fourth is deemed to have a high risk of bias [22]. A risk of bias summary is demonstrated in Figure 2 and Table 4.

### 3.2. Primary Outcomes

#### 3.2.1. Incidence of Incisional Hernia

**Figure 3:**
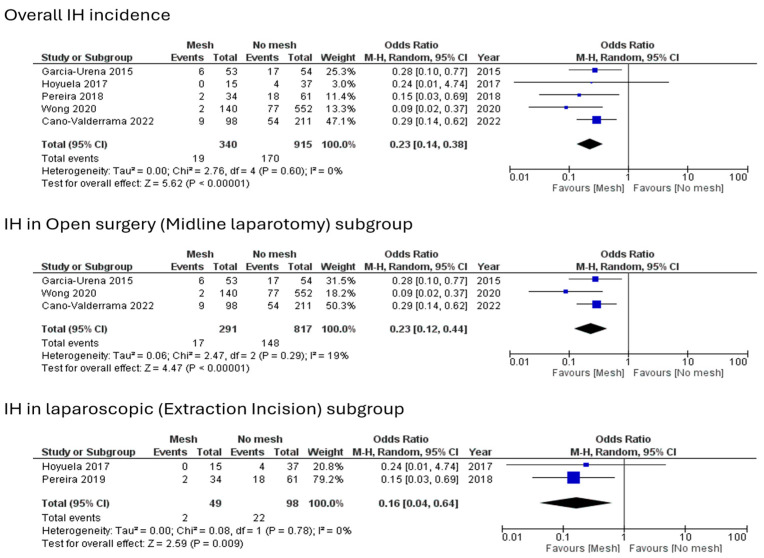
Forest plots for IH.

Five of the six studies evaluated the incidence of IH in their 1255 included patients. One hundred eighty-nine patients developed IH during the follow-up periods. Methods of assessment were clinical examination, computed tomography (CT) imaging or both, as clarified in Table 1. The pooled outcome shows that IH occurred significantly less in the mesh closure group with an odds ratio (OR) of 0.23, 95% CI of 0.14–0.38, and *p*-value of 0.00001. The studies had a low level of heterogeneity (I^2^ = 0%, *p*-value = 0.60), as shown in Figure 3.

Subgroup analysis of both open surgery and laparoscopic procedures shows significant outcomes. A total of 165 out of 1108 patients developed IH at their midline laparotomy incision following their open procedures. The pooled data favoured the mesh group with statistically remarkable differences (OR = 0.23, 95% CI = 0.12–0.44, and *p* = 0.00001). As for laparoscopic resections, IH at EI sites was found in 24 out of 147 patients with a statistical preference for mesh closure (OR = 0.16, 95% CI = 0.04–0.64, and *p* = 0.009) (Figure 3). The site, length, and outcome of EI were variable or not assessed/reported, as shown in Table 5.

#### 3.2.2. Evisceration Rate

**Figure 4:**
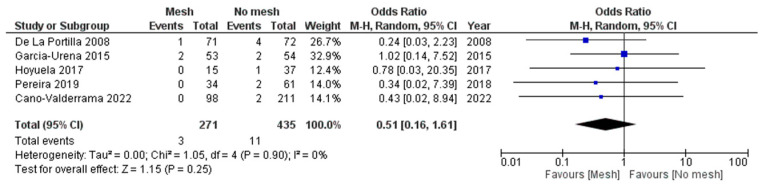
Forest plots for evisceration rate.

Only 14 patients had wound dehiscence early postoperatively. Although it was more in the non-mesh group, the discrepancy had no statistical value, with an OR of 0.51, 95% CI of 0.16–1.61, and a *p*-value of 0.25 [5,20,21,22,24]. The gathered studies showed a low level of heterogeneity (I^2^ = 0%, *p*-value = 0.90) (Figure 4).

The management of evisceration cases was not reported in the studies; therefore, a realistic estimate of this complication could not be evaluated.

### 3.3. Secondary Outcomes

#### 3.3.1. Surgical Site Infection (SSI)

**Figure 5:**
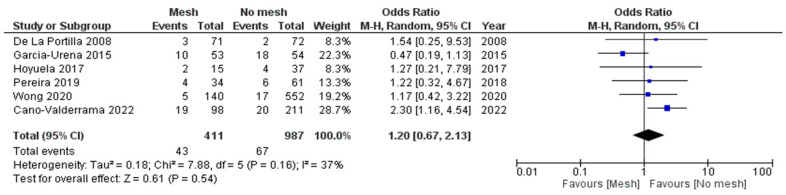
Forest plots for SSI.

Wound infection, or SSI, as a complication, was reported in all six studies, evidenced by 110 patients having had SSI. There was no notable difference between mesh and no-mesh groups (OR = 1.20, 95% CI = 0.67–2.13 and *p*-value =0.54), with a low level of heterogeneity among studies (I^2^ = 37%, *p*-value = 0.16), as clarified in Figure 5.

#### 3.3.2. Seroma Formation

**Figure 6:**
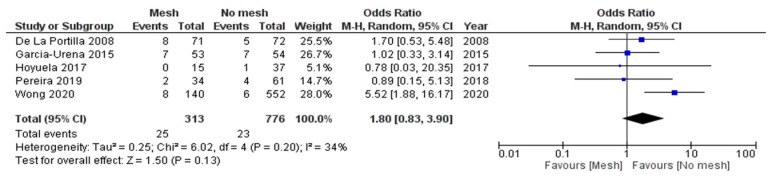
Forest plots for seroma formation.

Forty-eight patients developed seromas at surgical sites. The risk in both groups was comparable in the five reporting studies (OR is 1.80, 95% CI = 0.83–3.90, and *p*-value = 0.13) [5,20,21,22,23]. The difference was not statistically significant, and data heterogeneity remained low (I^2^ = 34%, *p*-value = 0.20) (Figure 6).

#### 3.3.3. Length of Hospital Stay (LOS)

**Figure 7:**
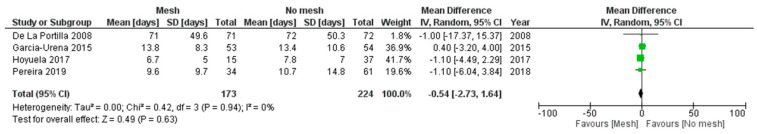
Forest plots for LOS.

LOS was only reported in four studies, with a total of 397 patients, and the pooled outcome was not favourable to any of the groups (mean difference −0.54, 95% CI = −2.73–1.64, and *p* = 0.63) [5,20,21,22] (Figure 7).

### 3.4. Sensitivity Analysis

The direction of the pooled effect size continued to be consistent when RR or RD was calculated for dichotomous variables. In addition, the leave-one-out analysis did not reveal any remarkable inconsistency with the original analysis.

## 4. Discussion

IH is a common postoperative complication of any abdominal surgery, with a reported incidence of 2–20% [25]. While the main risk factors are high BMI, previous abdominal surgeries, and impaired healing, the spectrum of contributing factors expands to include smoking, the presence of stoma, previous hernias, and flawed operative techniques. Unfortunately, even with surgical repair, recurrence remains high, up to 20% [3]. Due to both high occurrence and recurrence rates of IH, prevention is an important consideration in the management of patients undergoing abdominal surgery [26]. This, for sure, lessens the financial costs of recurrent hospital admissions due to symptomatic IH or additional surgeries to repair primary and recurrent IH [27]. Overall, most preventive measures do not require large resources [28]. Several measures can be adopted, including patient education and the use of appropriate suture, mesh, and abdominal closure techniques. Patients should be educated on the importance of maintaining a healthy weight, quitting smoking, optimising underlying health conditions, and avoiding heavy lifting and strenuous activity after surgery. It has been shown how obesity, uncontrolled diabetes, and alcohol intake can increase the costs of care required for patients with IH [27].

Techniques of closing laparotomy incisions have been refined over the years thanks to advancing technology and surgical research. Jenkins introduced the 4:1 rule when he used a ratio of 4:1 between suture and wound length (SL/WL ratio) [29]. A ratio of less than 4:1 was found to be associated with an increased hazard of IH. The large bite technique, where 1 cm bites of aponeurotic edge were taken at 1 cm gaps, was adopted to ensure a greater SL/WL ratio. This technique was vastly implemented until further research recommended a small-bite method, which was found to have a lower IH rate in the STITCH trial [30]. Since the invention of these methods, a slowly absorbable suture material has been in use. 

Although there have been numerous studies on the optimal techniques and materials for abdominal wall closure, many questions remain unanswered. For instance, there is uncertainty about the most appropriate method for closing laparotomy incisions in emergency situations and contaminated environments, as well as for closing non-midline laparotomies. Additionally, there is a lack of consensus on the best approach for closure in tricky situations or in patients at considerable risk of complications. This can be particularly challenging in emergency situations, where time is of the essence, and the risk of complications such as wound infections is high [31]. In such cases, the surgeon may have to prioritise speed over precision when choosing a closure technique, which could potentially increase the risk of complications in the long term. Moreover, closing laparotomies in contaminated environments can be challenging because of the increased risk of infection. Surgeons must consider using a closure technique that minimises the risk of contamination and promotes wound healing. Closure of non-midline laparotomies, as EI in laparoscopic resections, presents another challenge for surgeons. Such incisions are often made in areas where there is less muscular support, leading to a higher risk of hernia formation. Therefore, it is essential to consider the appropriate suture techniques and materials that can provide adequate support and reduce the risk of hernia formation.

In recent years, there has been an increasing interest in the potential prophylactic use of mesh to prevent the development of hernias in high-risk patients. There have been some promising results [32]. However, these studies have primarily focused on elective patients, and further research on the use of mesh in emergency situations is still needed. Undoubtedly, there are ongoing controversies on prophylactic mesh use. The increased operative time and costs of using a mesh may make this technique less appealing for surgeons and theatre management [33,34]. In addition, there have been safety concerns regarding mesh use in surgery, especially if it is used for prophylactic purposes that some may consider unnecessary overtreatment. These complications may include mesh migration, erosion to the bowel, chronic pain, bowel obstruction, and fistulae [35]. The risk of SSI is still thought to be higher when using a foreign body as a surgical mesh in surgery, although some reports mentioned no statistically notable risk [36]. Another potential adverse effect that has become widely known in media and legal reports recently is chronic pain. Despite its low risk with the laparoscopic approach and using low-weight meshes, it has been noted after mesh repair of ventral hernias [37]. For fairness, some of these complications remain relatively rare and less common than hernia recurrence and its implications when the mesh is not used in the repair. 

Recent reviews favoured the mesh closure when assessing patients having any abdominal surgery [38,39]. As for elective laparotomy, the evidence is reasonably sufficient in the literature showing the clear benefit of this technique in lessening IH risk following surgery. Mesh augmentation was reported to reduce IH incidence significantly following a short follow-up period of 12 months (1.5% in the mesh group versus 35.9% in the suture closure group) [40]. This effect was maintained up to five years of follow-up (5.1% vs. 46.8%) [41]. There was no notable difference in associated complications, apart from seroma formation being common with mesh use [40]. A systematic review of 11 RCTs and three prospective comparative studies demonstrated an 85% reduction in the occurrence of IH postoperatively compared to the conventional suture closure [42]. In contrast, the literature remains relatively patchy in terms of evaluating primary mesh closure of emergency laparotomy [43]. A significant proportion of laparotomies are currently conducted in emergency situations, a context that correlates with increased incidences of incisional hernias (IH) compared to those performed electively [44]. Therefore, the use of prophylactic mesh in emergency laparotomies is a topic of ongoing research [45].

This positive outcome is more evident in high-risk groups, such as patients with high BMI and connective tissue disorders. While most bariatric procedures are performed laparoscopically nowadays, some patients still undergo open operations [46]. A systematic review showed how mesh closure can lessen the risk of IH following bariatric surgery [47]. A study specifically focusing on AAA repair found that IH occurrence was three to nine times higher compared to other surgical procedures. This increased risk may be attributed to underlying connective tissue disorders associated with AAA [48]. In a Belgian randomised trial, retro-muscular mesh-augmented closure was safe and effective over a 2-year follow-up of patients who had abdominal aortic aneurysm repair [49].

While colorectal operations come with a high risk of IH and wound infections [50], mesh reinforcement of the abdominal wall has been used in stoma closure procedures, where 30% can have IH [51]. A 2020 RCT used a biological mesh on the stoma closure site and concluded a positive outcome in reducing IH and an acceptable safety profile [52]. Six other studies were pooled in a novel meta-analysis that supported the considerable reduction of IH when mesh was used to reinforce closure after stoma reversal [53]. Hence, this bright preventive technique should be evaluated closely for potential use in colorectal surgical procedures.

Our systematic review and meta-analysis mainly evaluate the efficacy of primary mesh closure in open and laparoscopic colorectal surgery. The results of the included studies provide compelling evidence for the risk reduction of IH when the mesh closure technique was employed during the closure of laparotomy or EI used in laparoscopic resections. Despite the inherent variations in closure techniques, mesh types, and positioning across these studies, mesh augmentation remained superior to conventional suture closure in all cases, even in subgroup analysis. Pooling the outcomes of these studies revealed that the mesh reinforcement technique has a satisfactory safety profile. Notably, there was no statistically significant difference in potential adverse effects, such as SSI, seroma formation, evisceration, and LOS. This suggests that the use of mesh in the closure of abdominal incisions does not significantly increase the risk of complications when compared to conventional suture closure.

The consistent and positive outcomes observed in these studies align with the reported results in numerous publications found in the literature [42,43,45]. This reinforces the reliability and validity of the findings, adding further weight to the argument in favour of using mesh reinforcement techniques during abdominal closure procedures. As a result, it can be concluded that adopting mesh closure techniques, regardless of the specific variations in technique or mesh type, is a viable and advantageous approach to reduce the risk of incisional hernias and improve patient outcomes.

This review included six studies, and numerical data were pooled into the meta-analysis, as demonstrated in the results section. Although the characteristics of the studied populations in terms of demographics, BMI, and associated comorbidities are comparable (Table 1), the methodologies followed are different. The examined populations are mainly European (Spanish), apart from one Australian study [23]. Two of the studies are RCTs [5,20], providing level 1 evidence, whereas the other four are cohort studies [21,22,23,24]. This demonstrates the need for more randomised trials of high-quality design to boost the reliability of the drawn conclusions. The included studies show a degree of heterogenicity. Half of them included patients who had both emergency and elective surgeries [5,20,23], while the other half investigated only elective colorectal procedures [21,22,24]. Although using a non-absorbable mesh in an onlay position is dominant in these studies, there are minor differences in the surgical techniques adopted in both suture and mesh closures, as clarified in Table 3. The follow-up timeframes also varied between studies, with one study having only a 30-day follow-up [20] and another having followed-up patients for a median of 13 months [22]. It is plausible to consider that the given time frame might not be adequate for accurately assessing the progression of complications. It is worth noting that the complexity of IH and its potential complications might require a longer observation period to gain a comprehensive understanding of its development.

This review has a few limitations, some of which have been clarified beforehand. The dataset remains limited in size, with one study not reporting on IH development [20]. Among the six studies that were included, four are comparative studies in nature, with three being retrospective [22,23,24] and one being prospective [21]. This introduces a substantial risk of bias in participant selection and leads to considerable divergence among the studies. Variations in closure techniques, mesh fixation, and placement, defining high-risk patients, and the potential presence of peritoneal contamination or soiling across each study could potentially affect the outcomes. In addition, the studies did not separate the reported outcomes of oncological and non-oncological conditions hence, any potential effect of cancer diagnosis could not be independently measured.

Another point that was not clear is the evisceration event and its management, surgically or conservatively. The reoperation rate in the early postoperative period was only reported in three studies, with one case of mesh infection/hematoma requiring mesh removal and replacement in one study [24] and three similar cases in another study requiring mesh removal [23]. In a third study evaluating the EI [21], mesh infections and hematomas were treated conservatively, and reoperation was only performed in two cases for suture dehiscence of the port site and intestinal obstruction, in which EI was not manipulated. Clearly, the evisceration events differ from reoperations; hence, detailed clarification of managing eviscerations and rate/indications of reoperations could not be elaborated due to the missing related information in most of the included studies.

Regarding the impact of EI, one of the two relevant studies used mesh closure in midline EI [22], while the other implemented mesh augmentation in both vertical and transverse incisions [21]. Interestingly, only one study reported the length of EI, which was 4–5 mm larger in the no-mesh group [21]. Overall, the prophylactic effect of mesh closure was maintained regardless of the EI site, as detailed in Table 5. There was no clarification on whether the length of EI could have a potential impact. This is a major consideration that could influence surgeons with certain preferences to implement the proposed technique.

Additionally, vital aspects like wound healing, persistent pain, aesthetic results, and long-term follow-up data are necessary to address patients’ expectations of this proposed technique. Furthermore, unifying the diagnosis of IH by using objective radiological diagnosis to detect subclinical hernias that may manifest a few years later can add more accuracy to the main primary outcome assessed in these studies. These points provide adequate perspectives for future research to explore.

Future research should focus on unifying the criteria of patients at higher risk and refining the mesh closure technique. For practicality, this technique has been proposed for high-risk patients to deliver the most cost-effective and effective treatment targeted to those who could benefit from it. Clearly, defining patients at risk and using risk calculators could enhance the patient selection for such an aggressive preventive strategy. Having uniform risk criteria for developing IH will encourage surgeons to offer such techniques to certain patients and have an objective discussion with them through weighing risks and benefits while clarifying the suitability of the available meshes in the market and the ideal mesh position for this recently proposed technique. Another valid point is the additional operative time required for mesh placement during prophylactic mesh augmentation typically falls within the range of ten to twenty minutes [34]. This approach not only facilitates the integration of prophylactic mesh placement into surgical practices but also ensures consistent outcomes and improves overall patient care [33]. To enhance its effectiveness for different patient populations, it is crucial to undertake further analysis of measures such as quality of life (QoL), return to work rates, assessments of chronic pain, and overall functionality [54]. By delving deeper into these aspects, surgeons can gain a better understanding of how to improve patient outcomes. This will enable them to develop clinical decision-making tools and guidelines that can be tailored to an individual’s specific risk factors, comorbidities, and anticipated postoperative functionality.

## 5. Conclusions

Undoubtedly, prophylactic mesh reinforcement has emerged as an effective approach to reducing incisional hernias, particularly in high-risk patients. In colorectal surgery, the reduction in IH with this technique is clear, with no significant associated adverse events; however, the balance between risks, benefits, and costs needs to be elaborated. Ultimately, further high-quality randomised trials would add more reliable weight to the results. There is still variability in the adoption of prophylactic mesh closure, although high-risk groups are well-known. Standardising the criteria will encourage surgeons to consider this technique in calculated discussions with patients.

## Figures and Tables

**Table 1:** Characteristics of the included studies.

Study/Design/Country	Population	Inclusion and Exclusion Criteria	Surgical Technique and Type of Mesh	Follow-Up Months	Primary Outcome	IH Assessment
**De La Portilla 2008 [20]** RCT Spain	No-mesh: 72 Mesh: 71 **Cancer diagnosis**: 80 (56%)	**Inclusion criteria**: All laparotomy incisions and mixed emergency and elective colorectal surgeries. **Exclusion criteria**: <18-year-old.	**No-mesh**: Mass closure with continuous Poly-p-dioxanone suture. **Mesh**: Same technique plus Supra-aponeurotic Polyglycolic mesh	1	**Evisceration** (Midline laparotomy) **No-mesh group**: 4/72 (5.6%) **Mesh group**: 1 (1.4%)	Clinical examination
**Garcia-Urena 2015 [5]** RCT Spain	No-mesh: 54 Mesh: 53 **Cancer diagnosis**: No-mesh 39/54 (72%) Mesh 45/53 (85%)	**Inclusion criteria**: Midline laparotomy incision, mixed emergency and elective colorectal surgeries. **Exclusion criteria**: Previous IH, haemodynamically unstable, carcinomatosis.	**No-mesh**: continuous with slowly absorbable suture poly-4 hydroxybutyrate. **Mesh**: Same above technique plus onlay large-pore polypropylene mesh	24	**IH** (Midline laparotomy) **No-mesh group**: 17/54 (31.5%) **Mesh group**: 6/53 (11.3%)	CT scan
**Hoyuela 2018 [21]** Prospect-ive cohort Spain	No-mesh: 37 Mesh: 15 **Cancer diagnosis**: 100%	**Inclusion criteria**: Vertical or transverse assistance incision for elective laparoscopic-assisted oncological resections, BMI > 25. **Exclusion criteria**: Emergency cases, conversion to open surgery, previous abdominal wall mesh.	**No-mesh**: Absorbable suture for peritoneum and continuous, slowly absorbable suture for the sheath. **Mesh**: Same above technique plus Retro-fascial pre-muscular sublay polypropylene mesh	22.3	**IH** (Extraction incision): **No-mesh**: 4/37 (10.8%) **Mesh**: 0/15 (0%) **Evisceration** (Extraction incision): **No-mesh**: 1/37 (2.7%) **Mesh**: 0/15 (0%)	Clinical examination + CT scan
**Pereira 2019 [22]** Retrospe-ctive cohort Spain	No-mesh: 61 Mesh: 34 **Cancer diagnosis**: 100%	**Inclusion criteria**: Elective Laparoscopic colorectal cancer surgery. **Exclusion criteria**: Previous open surgery, conversion to open surgery.	**No-mesh**: Continuous PDS loop. **Mesh**: onlay mesh of polyvinylidene fluoride.	13	**IH** (Extraction incision): **No-mesh**: 18/61 (29.5%) **Mesh**: 2/34 (5.9%) **Evisceration** (Extraction incision): **No-mesh**: 2/61 (3.3%) **Mesh**: 0/34 (0%)	Clinical examination ± CT scan
**Wong 2020 [23]** Retrospect-ive cohort Australia	No-mesh: 552 Mesh: 140 **Cancer diagnosis**: 486/662 (73%)	**Inclusion criteria**: Midline laparotomy incision, mixed emergency and elective colorectal surgeries.	**No-mesh**: Continuous with 1 Nylon suture, 1cm bite/gap. **Mesh**: Same technique plus onlay Polypropylene mesh	33	**IH** (Midline laparotomy) **No-mesh**: 77/553 (13.9%) **Mesh**: 2/140 (1.4%)	Not mentioned
**Cano-Valderra-ma 2022 [24]** Retrospect-ive cohort Spain	No-mesh: 211 Mesh: 98 **Cancer diagnosis**: No-mesh 179/211 (85%) Mesh 75/98 (77%)	**Inclusion criteria**: Elective laparotomy for colorectal surgery. **Exclusion criteria**: Laparoscopic surgery, follow up < 1 month.	**No-mesh**: Single-layer running suture. **Mesh**: Same above technique plus polypropylene mesh (Sublay in 9 cases and onlay in 79 cases)	22	**IH** (Midline laparotomy) **No-mesh**: 54/211 (25.6%) **Mesh**: 9/98 (9.2%)	CT scan ± Clinical examination

RCT: randomised controlled trial, PDS: polydioxanone suture, BMI: body mass index, IH: incisional hernia.

**Table 2:** Summary of results.

	Mesh Closure	No-mesh Closure	Odds Ratio (OR)	95% CI	*p*-Value
**Primary outcomes:**					
Incisional hernia (Midline Laparotomy)	17/291 (5.8%)	148/817 (18%)	0.23	0.12-0.44	* <0.00001
Incisional hernia (Extraction site)	2/49 (4%)	22/98 (22.4%)	0.16	0.04-0.64	* 0.009
Evisceration	3/271 (1.1%)	11/435 (2.5%)	0.51	0.61-1.61	0.25
**Secondary outcomes:**					
Surgical site infections	43/411 (10.5%)	67/987 (6.8%)	1.20	0.67–2.13	0.54
Seroma formation	25/313 (8%)	23/776 (3%)	1.80	0.83–3.90	0.13
Length of hospital stay	Mean difference = −0.54			−2.73–1.64	0.63

* Significant result.

**Table 3:** Baseline characteristics of the included population.

Study	Age [mean ± SD or Median (Range)] (years)	Gender Male: Female	BMI (Average/Mean ± SD)	Comorbidities
**De La Portilla 2008 [20]**	Mesh: 66.2 ± 12.53 No-mesh: 63.1 ± 15.6	Mesh: 41:30 No-mesh: 39:33	NA	Mesh: DM = 7, Steroids = 2, Respiratory disease = 5, Obesity = 2 No-mesh: DM = 4, Steroids = 4, Respiratory disease = 4, Obesity = 0
**Garcia-Urena 2015 [5]**	Mesh:65.6 ± 13.3 No-mesh: 61.46 ± 15.6	Mesh: 31:22 No-mesh: 33:21	Mesh: 24 No-mesh: 22	Mesh: DM = 18, Immuno-compromised = 6, Smoking = 5 No-mesh: DM = 9, Immuno-compromised = 5, Smoking = 9
**Hoyuela 2018 [21]**	Mesh: 76.4 ± 11 No-mesh: 71 ± 11	Mesh: 10:5 No-mesh: 23:14	Mesh: 27.8 ± 2 No-mesh: 28.9 ± 2	Mesh: DM = 4, Respiratory disease = 2 No-mesh: DM = 10, respiratory disease = 4
**Pereira 2019 [22]**	Mesh: 72.4 ± 10.9 No-mesh: 69.3 ± 12.5	Mesh: 17:17 No-mesh: 40:21	Mesh: 30.2 ± 5.6 No-mesh: 26.8 ± 4.4	Mesh: DM = 10, COPD = 10, Obesity = 19, Immuno-compromised = 3 No-mesh: DM = 13, COPD = 7, Obesity = 7, Immuno-compromised = 2
**Wong 2020 [23]**	All patients: 65 (20–96)	All patients: 55.5%:44.5%	NA	NA
**Cano-Valderrama 2022 [24]**	Mesh: 73.4 ± 11.3 No-mesh: 69.6 ± 11.9	Mesh: 53:45 No-mesh: 113:98	NA	Mesh: DM = 33, COPD = 8, Obesity = 27, Steroids = 4, Malnutrition = 9 No-mesh: DM = 41, COPD = 18, Obesity = 52, Steroids = 8

SD: Standard deviation, BMI: Body mass index, NA: not available, DM: diabetes mellitus.

**Table 4:** The methodological quality of the observational studies assessed with the Newcastle-Ottawa scale. * Indicates one point.

Study	Hoyuela, 2017 [21]	Pereira, 2019 [22]	Wong, 2020 [23]	Cano-Valderrama, 2022 [24]
Representativeness of the exposed cohort	*	*	*	*
Selection of the non-exposed cohort	*	*	*	*
Ascertainment of exposure	*	*	*	*
Demonstration that outcome of interest was not present at start of study	*	*	*	*
Comparability of cohorts based on the design or analysis controlled for confounders	*			
Assessment of outcome	*	*	*	*
Was follow-up long enough for outcomes to occur	*		*	*
Adequacy of follow-up of cohorts	*	*	*	*
Total	8	6	7	7

**Table 5:** Extraction incision (EI) used in the 2 relevant studies:.

Studies		Midline EI		Lateral Transverse		Low Transverse (Suprapubic)		EI Size (cm)
		Mesh	No-Mesh	Mesh	No-Mesh	Mesh	No-Mesh	
**Hoyuela 2018 [21]**	Number	3	7	4	11	8	19	Mesh: 5.8 ± 1
	IH incidence	0	3 (43%)	0	1 (9%)	Not performed		No-mesh: 6.2 ± 2
**Pereira 2019 [22]**	Number	34	61	Not performed			87	Not reported
	IH incidence	2 (5.9%)	18 (29.5%)				3 (3.4%)	

## Data Availability

The authors confirm that the data supporting the findings of this study are available within the article and the Supplementary Material. The raw data are available from the corresponding author on request.

## Supplementary Materials

The following supporting information can be downloaded at: https://www.mdpi.com/article/10.3390/jcm13226980/s1, Supplementary S1: PRISMA 2020 Checklist.
